# Testicular metastasis 9 years after resection of primary descending colon cancer with simultaneous pulmonary metastasis: a case report

**DOI:** 10.1186/s40792-023-01684-x

**Published:** 2023-06-13

**Authors:** Daishi Yoshimura, Yuki Sekido, Hidekazu Takahashi, Tsuyoshi Hata, Atsushi Hamabe, Takayuki Ogino, Norikatsu Miyoshi, Mamoru Uemura, Hirofumi Yamamoto, Yuichiro Doki, Hidetoshi Eguchi

**Affiliations:** Department of Gastroenterological Surgery, Graduate School of Medicine, Osaka University, Yamadaoka 2-2, Suita-Shi, Osaka, 565-0871 Japan

**Keywords:** Colorectal cancer, Testicular metastasis, Heterochronic recurrence

## Abstract

**Background:**

Metastatic testicular cancer is rare. In particular, primary colorectal cancer rarely metastasizes to the testes. This study reports a case of testicular metastasis recurrence 9 years after the resection of a primary colorectal cancer and a simultaneous metastatic lung tumour.

**Case presentation:**

A 69-year-old man underwent a laparoscopic left hemicolectomy for descending colon cancer. Preoperative computed tomography revealed a solitary left lung mass. Postoperative chemotherapy reduced the size of the lung mass, and 6 months after the primary resection, the patient underwent a left upper segmentectomy. Based on the pathological examination, he was diagnosed with pulmonary metastasis from colorectal cancer. After four courses of adjuvant chemotherapy, the patient was recurrence-free. However, 9 years and 6 months after the primary resection, he complained of discomfort in his left testicle. Physical examination revealed a left testicular mass. Since a malignancy was not excluded via imaging, left testicular resection was performed to confirm the diagnosis. The pathological diagnosis was testicular metastasis from colorectal cancer. The patient was followed up without medication, and remained healthy, without recurrence, 11 months postoperatively.

**Conclusions:**

It is important to follow up with testicular metastasis in mind, although it is rare.

## Background

Metastatic testicular cancer rarely occurs, in two large autopsy series, metastasis of the testis have been reported to approximately 0.06% of autopsy specimens [1, 2]. In addition, in a retrospective autopsy study of adult male with solid malignant tumour, 0.68% of autopsy specimens were shown to have metastatic deposits within the testis [3]. The most frequent primary lesion that metastasizes to the testis is prostate cancer, which accounts for 29–35% of metastatic testicular tumours. In contrast, testicular metastases from colorectal cancer (CRC) comprise 7–9% of all metastatic testicular lesions [4, 5]. This study reports a case of testicular metastasis recurrence 9 years after the resection of a primary CRC and a simultaneous metastatic lung tumour.

## Case presentation

A 59-year-old man underwent chest radiography during a health check-up at his workplace.

**Fig. 1:**
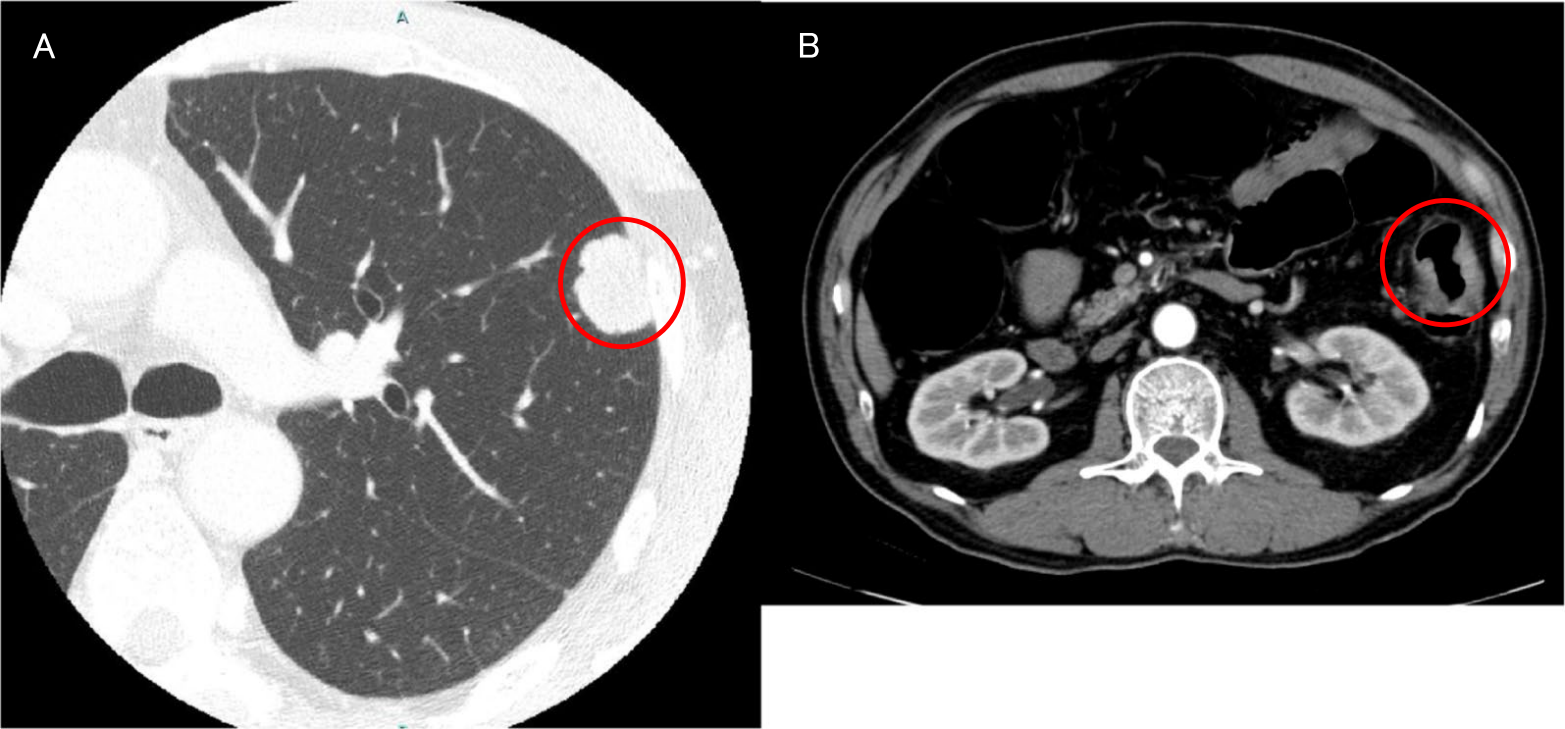
Computed tomography (CT) findings. **A** Plain chest CT revealed a solitary well-defined mass, measuring approximately 30 mm, in the superior region of the upper lobe of the left lung (red circle). **B** Abdominal contrast-enhanced CT revealed a subtotal mass with a contrast effect in the centre of the descending colon (red circle)

**Fig. 2:**
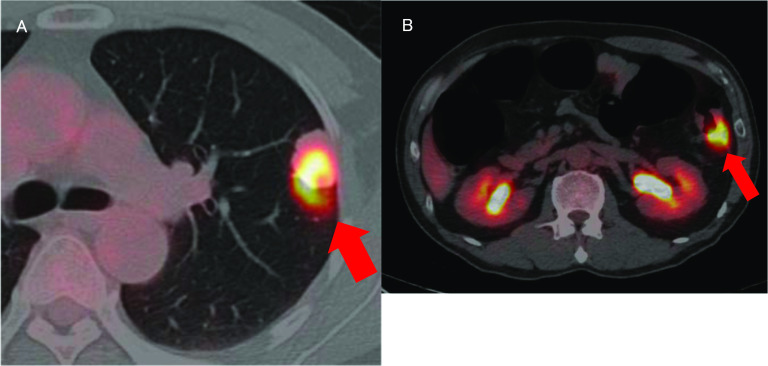
Positron emission tomography (PET)–CT findings. **A** PET–CT revealed a standardized uptake value (SUV) max of 9.2 fluorodeoxyglucose (FDG) accumulation in S3a of the left upper lung lobe (red arrow). **B** PET–CT revealed a SUV max of 9.8 FDG accumulation in the descending colon (red arrow)

**Fig. 3:**
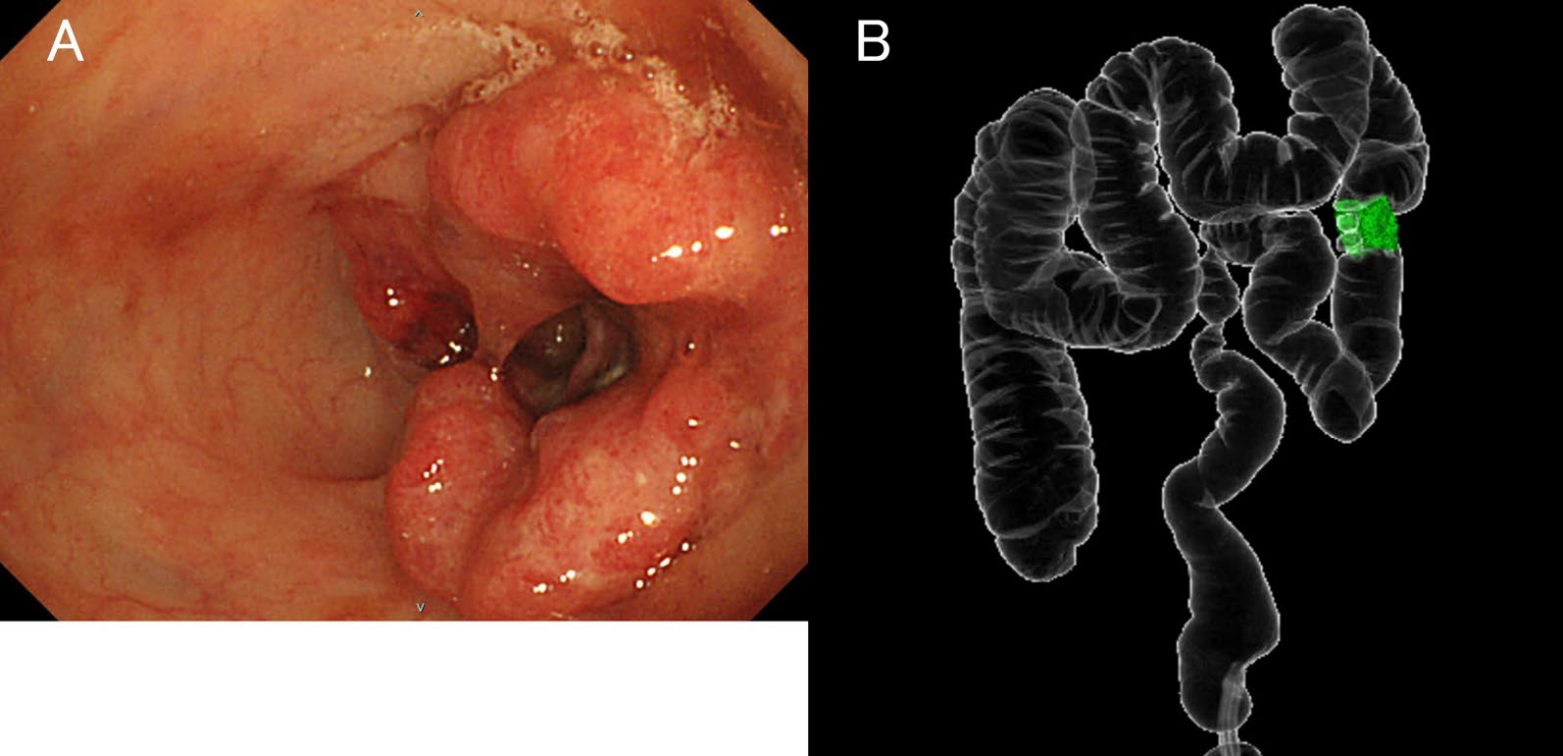
Colonoscopy and CT–colonography findings. **A** Colonoscopy revealed a semicircular type 2 lesion in the middle segment of the descending colon. **B** CT colonography showed a lesion in the middle segment of the descending colon

**Fig. 4:**
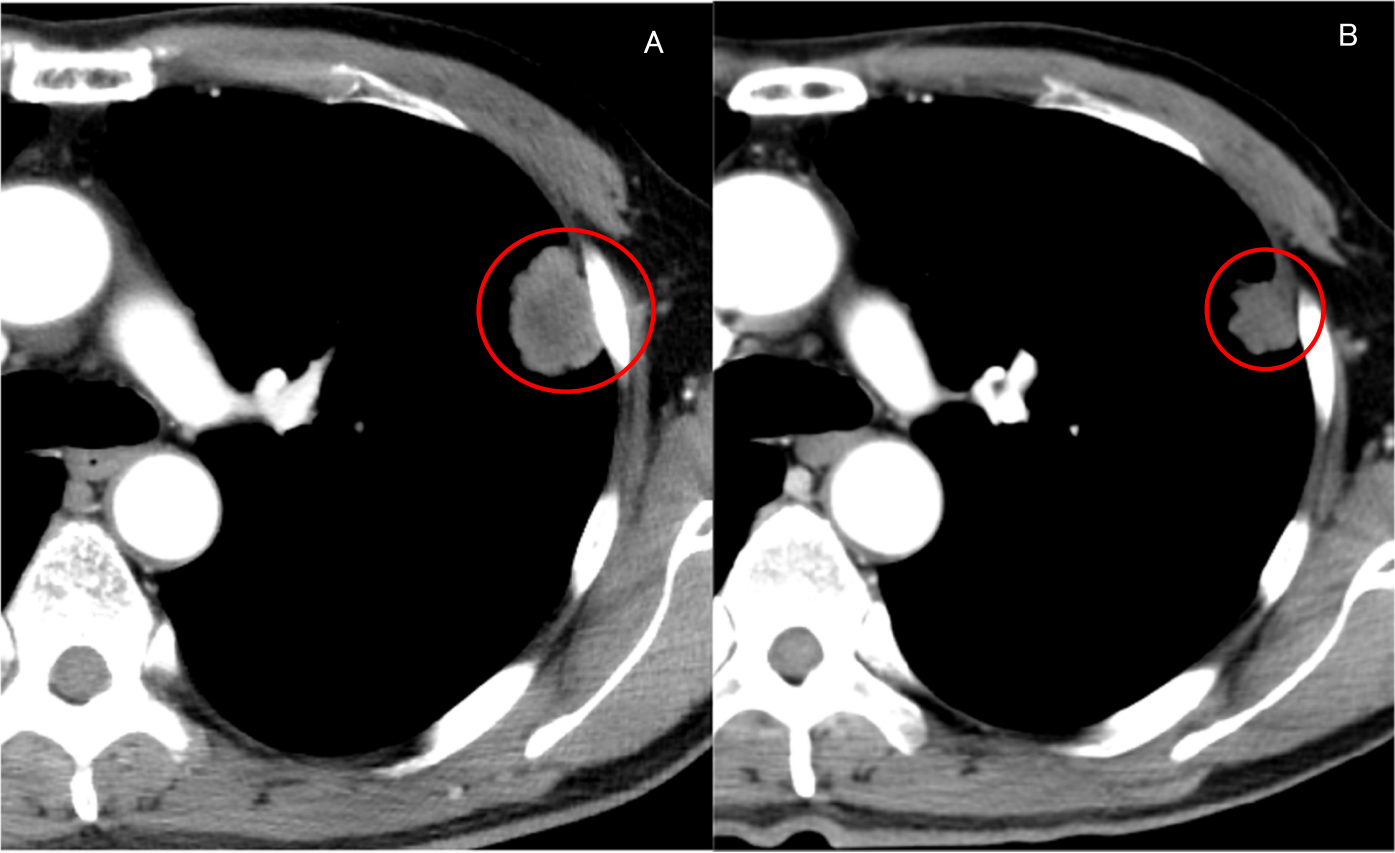
Shrinkage of a left lung mass after chemotherapy. **A** Mass in the superior region of the upper lobe of the left lung before chemotherapy (red circle). **B** Size of the left lung mass was reduced after chemotherapy, consisting of tegafur/gimeracil/oteracil (S-1) combined with oxaliplatin and bevacizumab, for four cycles (red circle)

**Fig. 5:**
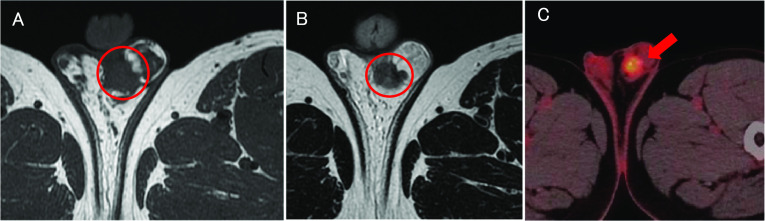
Magnetic resonance imaging (MRI) and Positron emission tomography (PET)–CT findings of the left testis. **A** MRI T1-weighted imaging revealed a 17 mm tumour with a low signal intensity on the cephalic side of the left testis (red circle). **B** MRI T2-weighted imaging revealed a 17 mm tumour with a low signal intensity on the cephalic side of the left testis (red circle). **C** PET–CT revealed a SUV max of 4.4 FDG accumulation, confined to the cephalic side of the left testis (red arrow)

**Fig. 6:**
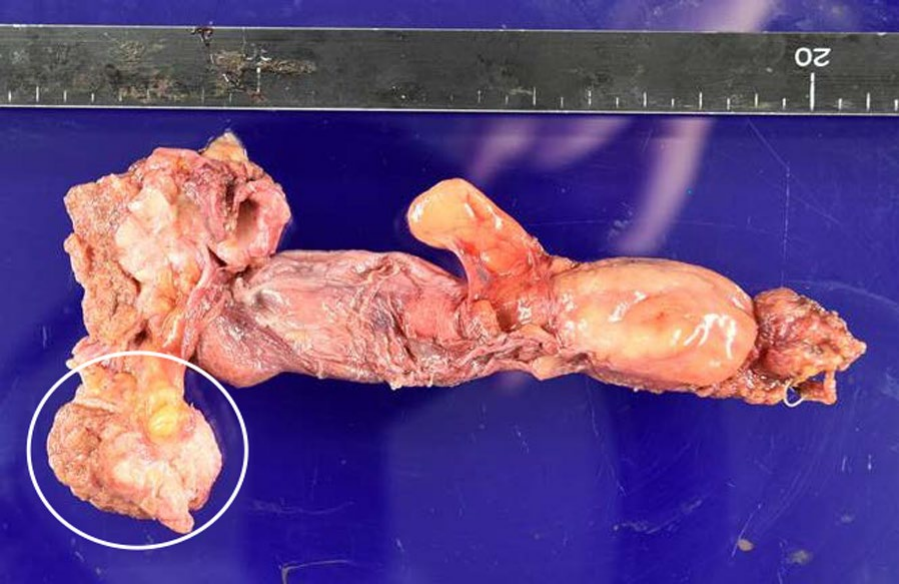
Macroscopic pathological findings of the left testis. The left testicle was removed via an orchiectomy (white circle)

**Fig. 7:**
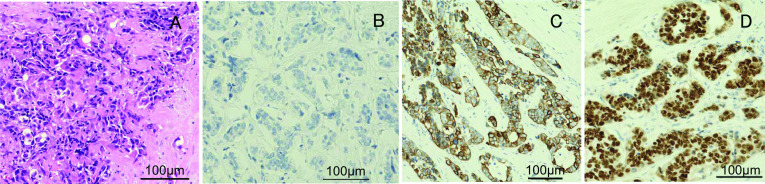
Microscopic pathological findings of the left testis. **A** HE staining showed atypical cells with irregular and swollen nuclei forming a distorted tubular structure. **B** On immunostaining, the tumour was negative for CK7. **C** On immunostaining, the tumour was positive for CK20. **D** On immunostaining, the tumour was positive for CDX2. HE: hematoxylin and eosin; CK7: Cytokeratin7; CK20: Cytokeratin20; CDX2: caudal-type homeobox 2

A nodular shadow on the lateral side of the left middle lung was detected. After a close examination by his local doctor, he was diagnosed with descending colon cancer and a simultaneous lung tumour. The patient was then referred to our department for further treatment. His medical history was unremarkable. Laboratory examinations showed mild anaemia only. The other laboratory data, including the tumour markers carcinoembryonic antigen (CEA) and carbohydrate antigen 19-9 (CA19-9), were essentially normal. Plain chest computed tomography (CT) revealed a solitary well-defined mass, measuring approximately 30 mm, in the superior region of the upper lobe of the left lung (Fig. 1A). Abdominal contrast-enhanced CT revealed a subtotal mass lesion with a contrast effect in the middle segment of the descending colon (Fig. 1B). Positron emission tomography (PET)–CT revealed a maximum standardized uptake value (SUV) max of 9.2 for fluorodeoxyglucose (FDG) accumulation in S3a of the left upper lung lobe (Fig. 2A) and an SUV max of 9.8 with FDG accumulation in the descending colon (Fig. 2B). Colonoscopy revealed a semicircumferential type 2 lesion in the middle segment of the descending colon (Fig. 3A). This finding was consistent with the CT colonography findings in the same area (Fig. 3B). A laparoscopic left hemicolectomy with regional lymph nodes dissection, including nodes around origin of inferior mesenteric artery, was performed. Based on the pathological examination, the patient was diagnosed with moderately differentiated tubular adenocarcinoma. According to the eighth version of the Union for International Cancer Control for International Cancer Control TNM classification for CRC, the patient had cStage IVA disease [6]. Subsequently, he received chemotherapy with tegafur/gimeracil/oteracil (S-1), combined with oxaliplatin (SOX) and bevacizumab for four cycles. Shrinkage of the left lung tumour was observed (Fig. 4). The patient underwent close surveillance during chemotherapy treatment to monitor the occurrence of additional lung metastatic lesions or other metastatic lesions. However, there was no evidence of new lesion formations during the observation period. Left upper segmentectomy was performed 6 months after the primary resection. Based on the pathological examination, most of the central part of the tumor was necrotic, and only a few atypical ducts of moderately differentiated adenocarcinoma showing a tendency to coalesce were observed only in the marginal part. In addition, the patient was diagnosed with metastatic lung tumours from CRC. The pathological diagnosis of CRC was well-differentiated adenocarcinoma > moderately differentiated adenocarcinoma, pT3N1a(1/9)M1a [PUL], pStage IVA, Ly1a, V0, PM0, DM0. He received adjuvant chemotherapy with SOX for four cycles after undergoing a pneumonectomy. Thereafter, he underwent follow-up for 5 years, during the follow-up evaluation, a contrast-enhanced CT and colonoscopy were performed, and the tumour markers were measured. No recurrence was observed during the follow-up evaluation. However, 9 years and 6 months after the primary resection, he experienced discomfort in the left testicle. Magnetic resonance imaging (MRI) revealed a tumour, measuring 17 mm, with a low signal intensity on T1- and T2-weighted imaging of the cephalic side of the left testis (Fig. 5A, B). PET–CT yielded a SUV max of 4.4 FDG accumulation, confined to the testis in the same area (Fig. 5C). The tumour markers, particularly the human chorionic gonadotropin, fetoprotein, CEA, and CA19-9, were within the normal ranges. In addition to malignancy, epididymal fibroma and epididymitis were raised as radiological differential diagnoses. Epididymal fibroma are mostly treated with radical orchiectomy, because preoperative diagnosis confirming the benign nature is difficult [7]. Therefore, a left orchiectomy was performed for diagnostic purposes. Based on the postoperative pathological examination and immunostaining results, the patient was diagnosed with testicular metastasis from CRC (Figs. 6 and 7). The carcinoma was located mainly in the testis and epididymis, but it also invaded the spermatic cord. He received adjuvant chemotherapy, consisting of capecitabine and oxaliplatin, but the medications were discontinued after one cycle due to the occurrence of epigastric discomfort after taking capecitabine. The patient was followed up without medication at his request. He is currently healthy, without recurrence, 11 months postoperatively.

## Discussion

Metastatic testicular cancers are rare, and the primary lesion seldom comes from the large intestine [1–5]. The details of the route of metastasis to the testis are unknown. Aside from the hematogenous and lymphatic routes, retrograde invasion of the ductus deferens, direct invasion along the spermatic cord, and peritoneal dissemination via congenital testicular effusion with patency of the sheath-like process were considered possible routes [8]. Since the pathological examination in this case showed lymphatic invasion at the time of the primary resection, lymphatic recurrence was considered likely. In addition, the pathology of the resected testis showed invasion of the spermatic cord, suggesting a slight possibility of direct invasion along the spermatic cord. A PubMed search for testicular metastases from CRC, using the keywords “Colorectal Cancer, Testicular Metastasis,” yielded 16 cases. Among these studies, ten involved heterochronic recurrences, and the details of these ten cases are summarized in Table 1[9–18]. The median age at the time of the primary resection was 64.5 years, while the median time from primary resection to recurrence was 24 months. Recurrence was independent of age, primary stage, primary localization, or histological type. According to the National Comprehensive Cancer Network (NCCN) Clinical Practice Guidelines in Oncology for colon cancer, patients with stage IV CRC without evidence of disease after curative-intent surgery and subsequent adjuvant treatment are recommended to undergo follow-up for 5 years. During the follow-up evaluation, a contrast-enhanced CT is performed, and the CEA is measured [19]. Among the 10 cases of heterochronic recurrence, some patients developed recurrence more than 5 years after the primary resection. The recurrence rate of CRC beyond 5 years after radical resection due to distant metastasis has reported to be 0.9% [20]. Liver (0.24%), lungs (0.22%) and peritoneum (0.09%) are the most common sites of recurrence due to distant metastases, in that order [21]. Even among Stage IV patients with distant metastases who have undergone radical resection, the most common sites of recurrence is reported to be the liver and lungs [22]. Accumulation of long-term follow-up results after radical resection in patients with stage IV CRC is needed, but testis is predicted to be an unlikely site of recurrence. With regard to histological types of CRC, approximately 95% of CRC in Japan are adenocarcinomas, most of which are well or moderately differentiated adenocarcinomas [23, 24]. Of the 10 cases of heterochronic recurrence, four showed a histological type other than well or moderately differentiated adenocarcinoma, which is clearly higher than the epidemiological ratio. This matter may have some relevance to the pathogenesis of testicular metastasis of CRC, but the number of cases in this study was small and further case accumulation is needed. Based on the results of the review, scrotal swelling was the most common diagnostic indicator of recurrence.

**Table 1:** Summary of heterochronic recurrence of testicular metastasis of colorectal cancer

Case Number	Age at primary resection	Time from first surgery to recurrence (months)	Pathological diagnosis of primary lesions			Localization of left and right of primary lesions*	Histological type^†^	Primary resection^‡^	Adjuvant	Diagnostic indicator of recurrence	Metastasis of peritesticular structures	Treatment	Outcome
			pT	pN	stage								
#1 [9]	74	12 M	4	0	II	Right/AC	Tub2	RHC + SBR + OR	+	Scrotal swelling and tenderness	Epididymis, Spermatic cord	Orchiectomy (for pain relief and diagnosis)	Died 2 months after orchiectomy
#2 [10]	64	12 M	3	1	III	Right/TC	Muc	RHC	+	Scrotal swelling	Epididymis, Spermatic cord	Started chemotherapy after right orchiectomy	ND
#3 [11]	51	24 M	ND	ND	ND	Left/DC	Tub2	Colon Resection	−	A small lump in the scrotum that has increased since the primary resection	Peritesticular soft tissue	Started chemotherapy after orchiectomy	ND
#4 [12]	60	8 M	3	2	III	Left/SC	Tub2	AR	+	Scrotal swelling and tenderness	Epididymis	Started chemotherapy	ND
#5 [13]	23	41 M	ND	ND	ND	Left/TC	Sig	LHC	ND	Scrotal swelling	Epididymis, Spermatic cord	Orchiectomy	Survived 9 months recurrence free after orchiectomy
#6 [14]	74	30 M	ND	ND	ND	Left/DC	ND	LHC	ND	Inguinal and testicular pain as well as a hydrocele testis	Epididymis, Spermatic cord	Orchiectomy (for pain relief and diagnosis)	ND
#7 [15]	71	60 M	3	1	III	Left/Rec	Tub2	LAR	+	Scrotal swelling	ND	Orchiectomy	Recurrence in lungs and liver 6 months after orchiectomy. Later died
#8 [16]	65	74 M	2	0	I	Left/Rec	Tub2	LAR	−	Testicular enlargement and pain during chemotherapy for recurrence (74 months after first surgery)	ND	Orchiectomy (for pain relief and diagnosis)	ND
#9 [17]	40	5 M	4a	2	III	Left/SC	Por	LHC	+	Pain in inguinal region, scrotal swelling	Spermatic cord	Chemotherapy and homeopathic treatment after orchiectomy	Died 6 months after orchiectomy
#10 [18]	70	24 M	3	1	III	Right/Appe	Por	RHC after Appendicectomy	+	Scrotal swelling	Spermatic cord	Started chemotherapy after orchiectomy	ND
Our case	59	108 M	3	1a	IVA^#^	Left/DC	Tub2 > Tub1	LHC	+	Discomfort in scrotum	Epididymis, Spermatic cord	Chemotherapy after orchiectomy	Survived 11 months recurrence free after orchiectomy

ND: Not Described

*AC: Ascending Colon, Appe: Appendix, DC: Descending Colon, Rec: Rectum, SC: Sigmoid Colon, TC: Transverse Colon

^†^tub2: Moderately differentiated adenocarcinoma, muc: Mucinous adenocarcinoma, por: Poorly differentiated adenocarcinoma, sig: Signet-ring cell carcinoma, tub1: well-differentiated adenocarcinoma

^‡^AR: Anterior Resection, LAR: Low Anterior Resection, LHC: Left Hemi Colectomy, OR: Omentum Resection, SBR: Small Bowel Resection, RHC: Left Hemi Colectomy

^#^Our case was classified by the 8th version of the Union for international cancer control (UICC) TNM classification of colorectal carcinoma. For other cases, no clear classification criteria were specified in the paper

## Conclusions

This study reported a case of testicular metastasis 9 years after the resection of primary descending colon cancer and pulmonary metastases. Patients with CRC rarely develop metastatic testicular recurrence 9 years after the primary resection.

## Data Availability

The data set, supporting the conclusions of this article, is available in the Springer Open.

## Abbreviations

CRC: Colorectal cancer
CEA: Carcinoembryonic antigen
CA19-9: Carbohydrate antigen 19-9
CT: Computed tomography
PET: Positron emission tomography
SUV: Standardized uptake value
FDG: Fluorodeoxyglucose
TNM classification: Tumour-node-metastasis classification
SOX: Tegafur/gimeracil/oteracil (S-1) combined oxaliplatin
MRI: Magnetic resonance imaging
